# Reefing Viability Index for Rigs-to-Reefs (R2R) in Malaysia

**DOI:** 10.1155/2020/4695894

**Published:** 2020-10-21

**Authors:** Mohd Hairil Mohd, Mohd Asamudin A. Rahman, Muhammad Nadzrin Nazri, Chun Hong Tan, Yuzwan Mohamad, Chuin Siew Lim, Baharim Mustapa, Hasrizal Shaari, Yii Siang Hii, Do Kyun Kim

**Affiliations:** ^1^Faculty of Ocean Engineering Technology and Informatics, Universiti Malaysia Terengganu, Kuala Terengganu, Terengganu, Malaysia; ^2^Faculty of Science and Marine Environment, Universiti Malaysia Terengganu, Kuala Terengganu, Terengganu, Malaysia; ^3^Institute of Oceanography and Environment, Universiti Malaysia Terengganu, Kuala Terengganu, Terengganu, Malaysia; ^4^Central Laboratory, Universiti Malaysia Terengganu, Kuala Terengganu, Terengganu, Malaysia; ^5^Universiti Malaysia Terengganu, Kuala Terengganu, Terengganu, Malaysia; ^6^Group of Marine Offshore and Subsea Technology, Newcastle University, Newcastle upon Tyne, UK; ^7^Graduate Institute of Ferrous Technology, Pohang University of Science and Technology, Pohang, Republic of Korea

## Abstract

Decommissioning of the offshore platform as an artificial reef, known as Rigs-to-Reefs (R2R), has become a sustainable approach for oil companies. The platform was reused to serve the underwater ecosystem as an artificial reef for a new marine ecosystem which helps to tackle food security issue. This paper presents the findings of the formulation of the reefing viability index to recognize an offshore region that can be used for R2R projects within the South China Sea. The combined effects of spatial data, numerical modelling, and geographic system (GIS) are proposed to study the relationship of spawning ground coral reefs, diversity, and planula larvae in the process of colonization to establish a map of the reef potential environment. Coral connectivity and spawning behaviour were studied to determine the possible source of coral seedling released during the spawning season, twice a year. A geographic reef viability index was established consisting of seven parameters which are coral larval density, pelagic larval length, sea currents, temperature, chlorophyll-a, depth, and substrate availability. The ocean hydrodynamic model was designed to resemble the pattern of larval scattering. By using the simulations and rankings, there were 95 (21%) sites which could probably be used for in situ reefing, whereas 358 (79%) sites were likely ideal for ex situ reefing. Validation of the viability index was carried out using media footage assessment of remotely operated vehicle (ROV).

## 1. Introduction

Oil and gas platforms have provided habitat for diverse fish and epibenthic organisms [1, 2]. The Rigs-to-Reefs program is conducted extensively in the Gulf of Mexico and has been used as a model for artificial reef systems in other countries [3]. The Rigs-to-Reefs program was developed in the mid-1970s for the oil and gas platforms around the Gulf of Mexico. The US Bureau of Ocean Energy Management (BOEM) came into a joint agreement with the Gulf States known as the “Rigs-to-Reefs” program. Oil/gas companies were allowed to donate platforms into the Rigs-to-Reefs program where they would remain on the shelf either in their original location, or they could be towed to a designated artificial reef site within the US Exclusive Economic Zone (EEZ). These structures are intended mainly to encourage the development of recreational and commercial fish populations [4].

**Figure 1:**
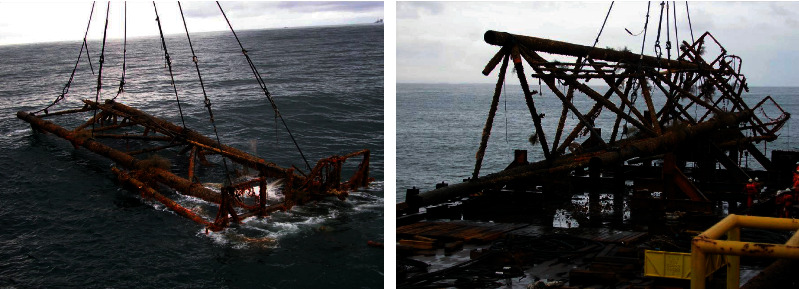
Baram-8 was rearranged into artificial reef structures [5].

In the South China Sea, there were several structures of oil rigs converted into artificial reefs. Seven of these rigs-to-reefs are located around the waters of Brunei Darussalam. Baram-8 is the only rig-to-reef structure in Malaysia. The Baram-8 oil platform was installed in August 1968, eight nautical miles away from Tanjung Baram, Miri. The structure collapsed in December 1975. After extensive studies, Baram-8 was salvaged and turned into an artificial reef at the end of 2004 (Figure 1). The conversion process of Baram-8 from an oil rig to the artificial reef was conducted on a floating barge at sea. Baram-8 was a collapsed single well with a three-legged jacket. The structure was cut into two sections and deployed at a shallower depth of 21 m with the top of the structure being 14 m from the sea surface.

After three months of relocation, Reef Check Malaysia reported large schools of juvenile jackfish around the converted structure of Baram-8. Large groupers were found in the lower part of the structure. There were also a variety of juvenile bannerfish, batfish, damselfish, fusiliers, snappers, sweetlips, coral trouts, and angelfish. The high diversity of fish during the early stage of rig-to-reef with established macrobenthic communities were good indicators of a sustainable artificial reef.

A survey conducted by Awang [5] in 2012 showed that the rig-to-reef Baram-8 had grown into a functional reef structure with a high abundance of fish, soft corals, and sponge. To date, there are no reports on the hard-coral composition at this site. An extensive survey should be carried out at the rig-to-reef Baram-8 for a comprehensive inventory of its inhabitants and overall productivity of the reef ecosystem.

Formulation of the reef viability index is proposed to estimate whether the current location of an offshore platform is suitable for reefing; thus, the platform owner can decide to choose the most suitable removal options for the decommissioning operation. The formulation involves data collection, modelling of coral larvae, and result integration using the geographical information system (GIS), ArcGIS. To ensure natural recruitment occurrence at the R2R site, factors such as the density of coral larvae, sea surface temperature (SST), chlorophyll, depth, and availability of substrate are taken into consideration. Most of the scleractinian corals release the gamete directly into the water column. Previous researchers used the particle tracking method coupled with a hydrodynamic-advection model to predict the trajectory of the gamete [6, 7]. This paper discusses about the results of the formulation for reef viability index established for Malaysia water in South China Sea. The geographic information system, ArcGIS, was used, which employs the spatial regression technique. Spatial data were analysed by layers; therefore, suitable reefing methods and area can be identified.

## 2. Methods

### 2.1. Hydrodynamic Model Validation

**Figure 2:**
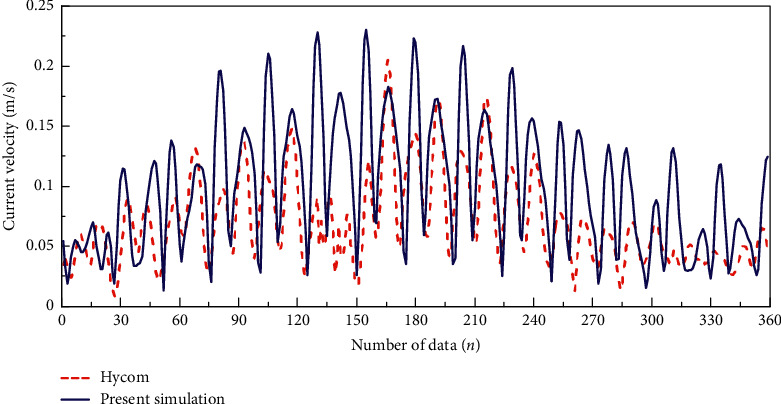
Current speed of Hycom versus present model (Mike 21).

**Figure 3:**
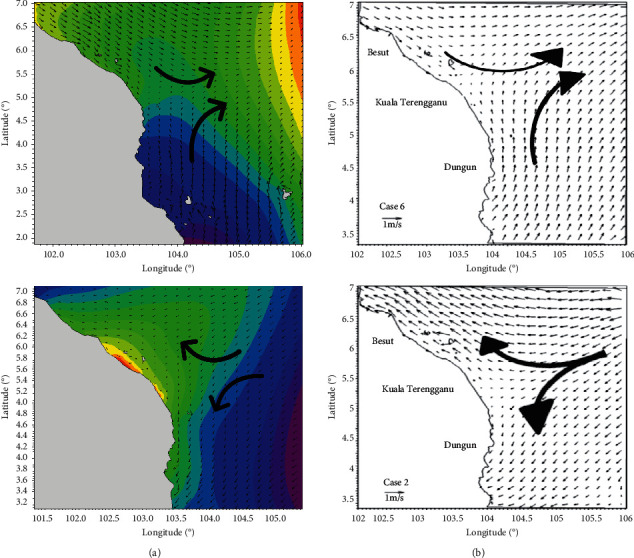
Comparison of major current direction for (a) present numerical model and (b) the model of Daud et al. [8].

This section reviews the performance and accuracy of the hydrodynamic model. By comparing Hycom data and model data, the RMSEs (root mean square errors) were calculated and discussed. RMSE is used to measure the differences of two data, predicted and observed (residual errors), to show their patterns of distribution. Lower values of RMSE contribute to better accuracy of the model. In this study, predicted data were obtained from Mike 21 and observed data was acquired from Hycom. The formula of RMSE is shown as follows:

(1) $$\begin{array}{c} \text{RMSE}=\sqrt{\frac{\sum_{i=1}^{n}{\left(p_{i}-\;o_{i}\right)}^{2}}{n}}, \end{array}$$

where, *n* is the number of data, *p*_*i*_ is the predicted data, and *o*_*i*_ is the observed data. Data were compared for 15 days, from 1st April 2016 to 15th April 2016 (Figure 2). There were 24 sets of data that represented 24 hours of each day. The RMSE of current speed was 0.05 m/s and showed very small residual errors. Visual comparison of the current direction was made with the study by Daud et al. [8] as demonstrated in Figure 3.

### 2.2. Bathymetry, Sea Surface Temperature (SST), and Surface Current

Coral settlement is directly affected by the environmental conditions in the targeted area. The bathymetric condition, sediment type, current circulation pattern and magnitude of the sea current, light penetration, and the state of nutrients are the main parameters of concern. As pointed out by Macreadie et al. [9], the R2R concept may not be beneficial when the rigs are in deep water. In this study, we redefined the depth limit for deep water is those waters that are deeper than 200 meters as most of the research in artificial reefs and rigs-to-reefs program are conducted in the water that are shallower than 200 meters [10]. Beyond 200-meter depth, it is difficult to predict the outputs of the R2R program.

**Figure 4:**
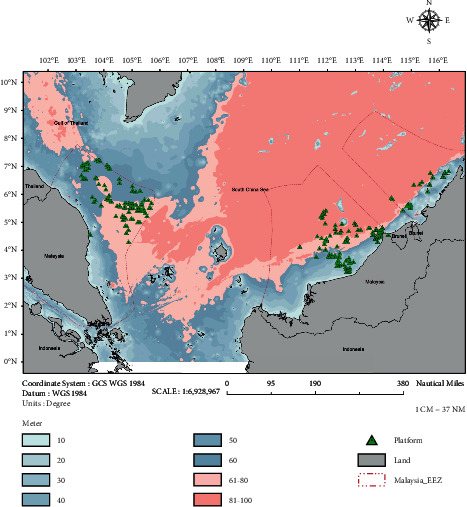
Bathymetry map of the water region in Malaysia.

The water depth has been categorized into six classes 0–10 m, 11–20 m, 31–40 m, 41–50 m, 51–60 m, and >80 m that corresponded to the potential of coral growth. Huston [11] reported that tropical coral reef growth is higher at shallow depths <30 m due to a higher intensity of light available than the coral at deeper depths, where the corals are expected to be rare or absent. The light intensity is strongly correlated with the water depth. Seawater absorbs specific wavelengths of visible light at a different depth. The long wavelengths of the light spectrum can penetrate to approximately 15 m (red spectrum light), 30 m (yellow spectrum light), and 50 m (orange spectrum light). These three light spectrums are essential for ocean productivity and photosynthesis process. The initial report by Grigg and Epp [12] suggested that approximately 30 m and 40 m are the critical depths for coral reefs in this word [12]. However, there are some reports revealing that coral reefs were found at depth >40 m [13–15] but such reefs were typically considered more as oddities than as essential components of coral reef ecosystems. A report by Grigg revealed that tropical corals do not grow well in the depths of over 50 meters [16]. The modern coral colonies below ∼50 m in the Au'au Channel (Hawaii) are unable to attach to the substratum and slowly collapse into the sand because of further biological and physical erosion [16]. The location of few platforms/offshore structures with the potential to be used in the R2R program was indicated with a green coloured triangle in Figure 4. The exact location of these platforms cannot be provided due to confidentiality of the information.

**Figure 5:**
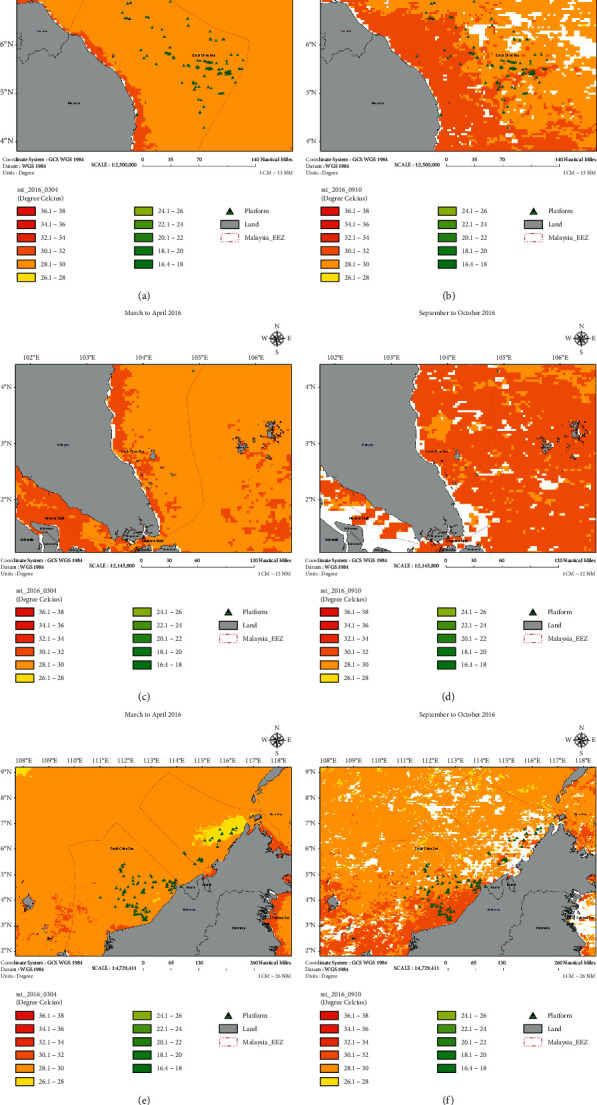
Sea surface temperature (SST) in Malaysia during March to April and September to October 2016. (a, b) North of Peninsular Malaysia. (c, d) South of Peninsular Malaysia. (e, f) Sabah and Sarawak.

**Figure 6:**
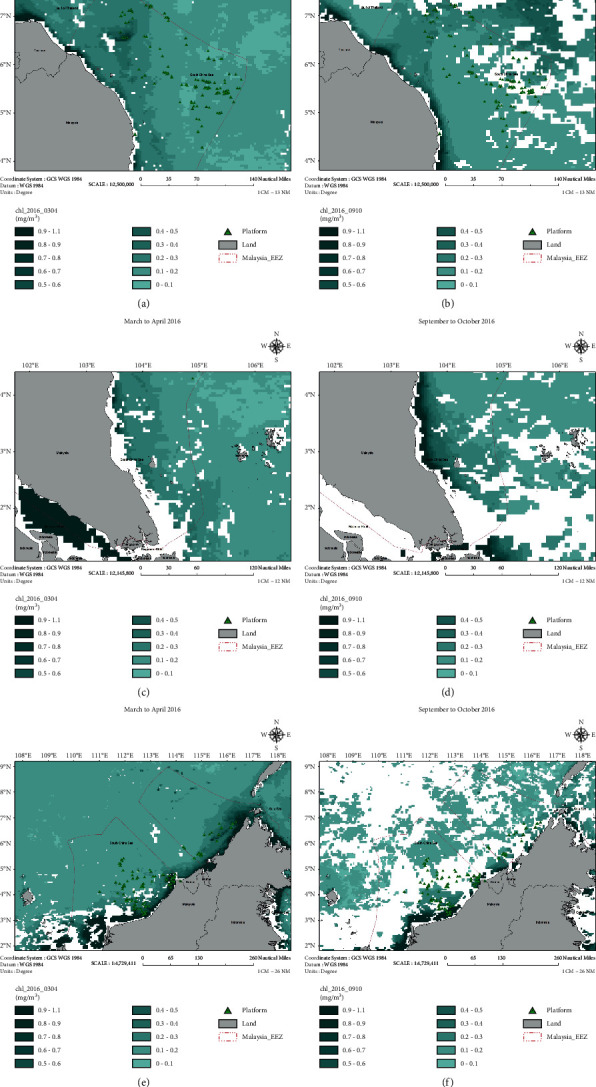
Chlorophyll-a content during September to October 2016. (a, b) North of Peninsular Malaysia. (c, d) South of Peninsular Malaysia. (e, f) Sabah and Sarawak.

**Figure 7:**
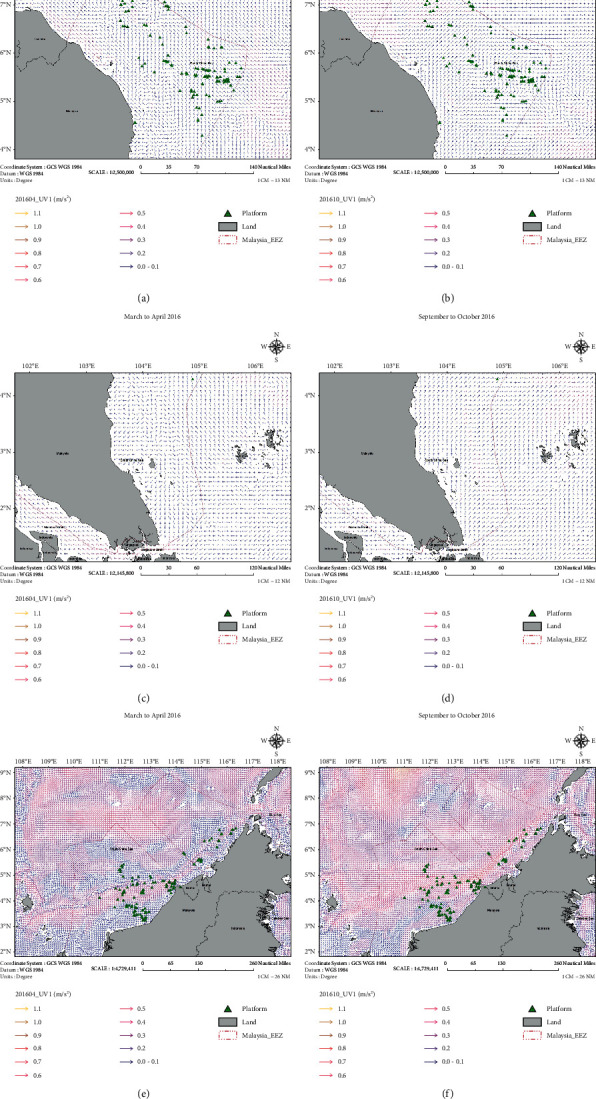
Surface current in Malaysia during March to April and September to October 2016. (a, b) North of Peninsular Malaysia. (c, d) South of Peninsular Malaysia. (e, f) Sabah and Sarawak.

The water column condition is an essential factor that enables to stimulate the growth of coral larvae. The physical parameters of the water column were established by using the ArcGIS based on the spawning period of the coral larvae, which is expected during September to October and March to April. At this stage, the sea surface temperature (SST) (Figure 5) and chlorophyll (Figure 6) images have been generated for both potential spawning periods. The SST was relatively high during the period of September to October 2016 as compared to March to April 2016. The SST value of approximately 33°C was recorded in a specific area, i.e., the coastal region of East Coast Peninsular Malaysia and Sarawak waters. Crabbe stated that high temperature (i.e., more than 32°C) would affect the growth of coral reefs due to the stress on coral polyps [17]. Meanwhile, chlorophyll concentration reflects the nutrient content in the water column. A high level of chlorophyll indicates high nutrient content in the water column and vice versa. The chlorophyll content was slightly higher during September to October 2016 than during March to April 2016. High nutrient content will disrupt the growth rate of coral reefs. The previous studies revealed that elevation of nutrient concentrations in the aquatic environment harmed the coral specimen [18, 19]. Hence, based on the SST and chlorophyll images, there is a high possibility that coral larvae will have a higher chance of growth during the spawning period of March to April than during September to October.  Figure 7 shows that the current was moving towards the coastal area during April 2016.

### 2.3. Coral Larvae Source Input

**Figure 8:**
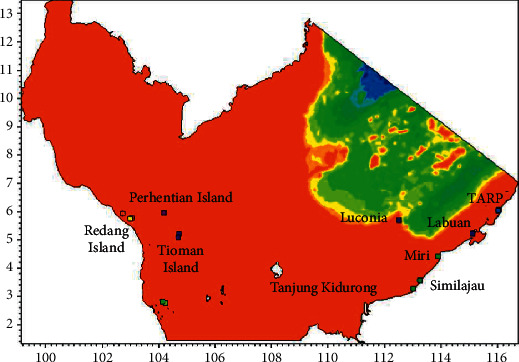
Coral spawning source locations.

The source sites of coral spawning were defined in the simulation by point sources as shown in Figure 8. The locations of coral seedling source are shown in Table 1, where 8 major source sites were used as coral larvae source locations in simulation runs. In Mike21-particle tracking module, three major inputs were considered to model the particles in this study: mass, coordinates, and number of particles released by time.

## 3. Results and Discussion

### 3.1. Coral Larvae Density

**Figure 9:**
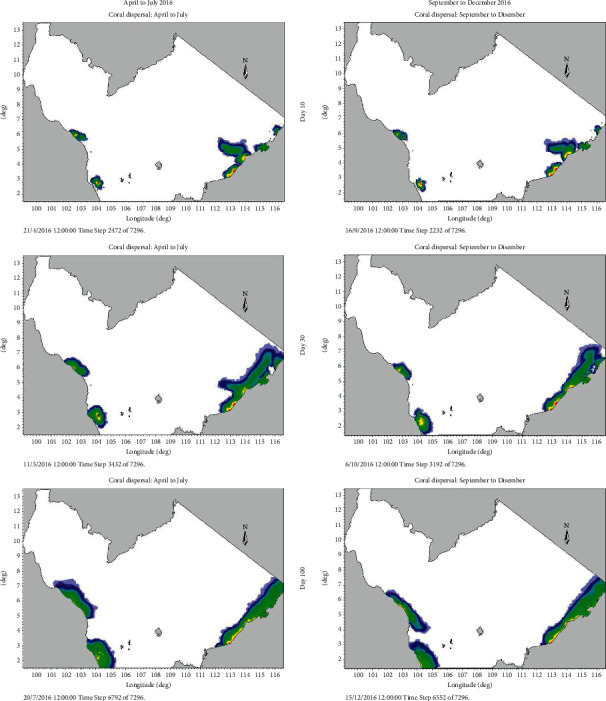
Coral particle dispersion simulation for April to July and September to December cycles in 2016. The pattern was captured on 10 days, 30 days, and 100 days, respectively.

The results are discussed in terms of coral larval dispersal direction and concentration area. Both seasons were compared side-by-side for every 10th day after coral spawning (Figure 9). On the 10th day after spawning, coral larvae from Perhentian and Redang islands were dispersed evenly in all directions during April to July and September to December cycles. While coral larvae from Tioman Island had dispersed evenly in all directions during April to July cycle, however, during September to December cycle, the majority of the larvae were spread to the south from its source. From Labuan and TARP, coral larvae dispersed towards the open sea and showed no connectivity like Miri and Labuan during August to December cycle. Coral larvae from Luconia source travelled southeast and connected with particle plume originated from Miri. This connectivity also occurred with coral larvae originated from Kidurong Cape and Similajau, Sarawak. On the 20th day after spawning, coral larvae from Perhentian and Redang islands were dispersed evenly in all directions towards north along the shoreline during April to July cycle. In contrast, during August to December cycle, larval dispersal did not indicate any significant direction. Coral larval dispersal during April to July did not show specific direction, but only growth outward into an evenly mass from its source, Tioman Island. Majority of coral larvae started to spread to the south of Tioman Island during September to December cycle. For East Malaysia, larval dispersal from Luconia, Kidurong Cape, Similajau, and Labuan was interconnected, leaving the larval plume originated from TARP unconnected as yet. The source from Luconia had wholly merged with coral larval dispersal.

Coral larvae circulated the islands south of Tioman Island during April to July, while during September to December cycle, coral larvae were concentrated along the shore of Johor. Source sites at Sabah and Sarawak on the 100th day showed no difference in terms of concentrated area and overall coral larval dispersal. Both cycles indicated that coral larvae had amassed at Kidurong Cape, Similajau, and Miri. This may be due to the deep-sea current (the offshore area from Miri to Sabah) of having its circulation, thus leaving the coral larvae to disperse in shallow water areas. However, at Sarawak, Kidurong Cape, and Similajau, the coral larvae did not travel far as the current might be circulating locally inshore and with minimal interaction with the offshore current.

### 3.2. Spatial Reef Viability Index

**Figure 10:**
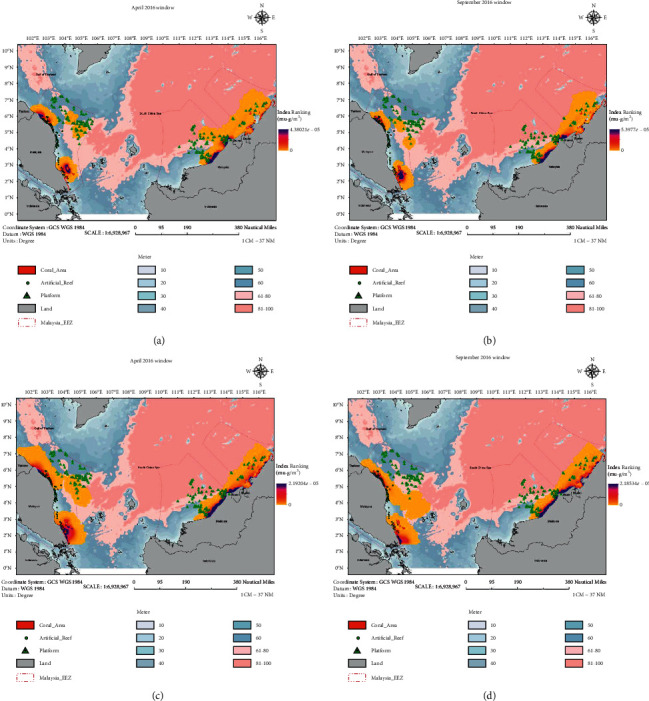
Integration of spatial data for April and September windows. The dispersion of the coral larvae shown at (a, b) 30 days and (c, d) 100 days after spawning event.

The reef viability index was calculated for 455 offshore structures in this study. The index was simulated at an interval of 10 days, for a 100-day period in two predominant windows in April to July and August to December.  Figure 10 shows the September window for 30 days and 100 days after spawning. Results revealed that 30 days after the coral spawning in April, the seedling had remained in its surrounding environment and did not affect the offshore environment. During the 30 days, the offshore environment did not have the opportunity to be exposed to the coral larvae. Hence, reefing viability is negative. Ultimately, the coral larvae in its planktonic stage would be drifted by sea currents towards the offshore environment. Within 100 days, some of the structure offshore would start to receive coral larvae, and the possibility of offshore reefing is there onwards increased from Miri during September to December, unlike April to July. This is due to the difference in current speed during both cycles. During the 30th day, there was no noticeable pattern change in the coral larval movement for all islands. At Sabah and Sarawak, coral larval dispersal had demonstrated interconnectivity between source sites during April to July cycle. On the 40th day, there was a pattern change of larval dispersal at Sabah-Sarawak during August to December cycle. The coral larval dispersal area was reduced and accumulated at the shoreline. As the coral larval dispersal pattern was constantly up to the 100th day in all locations, the discussion will be summarized after the 100th-day simulation diagram. For Redang and Perhentian islands, majority of the coral larvae headed to the north of their original site during April to July cycle. In contrast, during September to December cycle, the direction of the larval dispersal turned southeast. For Tioman Island, the general patterns of both sequences were the same. After 100 days of drifting, the coral larvae would be distributed via sea current. Most of the coastal environment alongside the east coast of Peninsular Malaysia, Sabah, and Sarawak became viable for reefing after 100 days in the April to July window. During the August window, similar to the April window, the first 30 days after coral spawning did not contribute to the viability of offshore reefing and the coral larvae were scattered around the source within a radius of few to a tenth of kilometres. The offshore environment received coral larvae 100 days after spawning. When compared to the April window, the high potential area during the August window is narrower.

**Figure 11:**
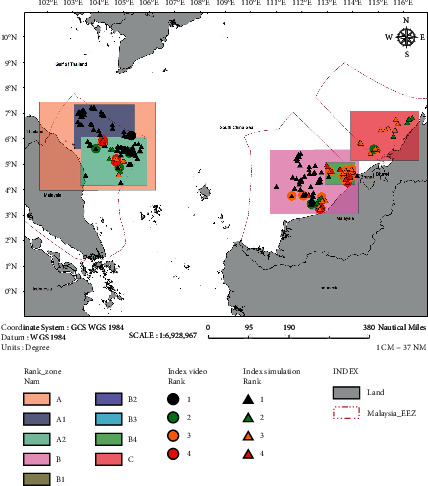
Reef viability ranking for the South China Sea region.

In this study, the simulation was conducted for seafloor as well as a simulated structure which had a headspace of 30 m clearance. Based on the outputs, on the seabed, 24 (5%) sites were identified as high potential sites (Rank 4). In comparison, 71 (16%) sites were identified as potential (Rank 3), 102 (23%) were classified as having low potential (Rank 2), and the remainder 256 (56%) sites were classified as unlikely (Rank 1). Also, 95 (21%) sites were found to be ideal for in situ reefing and 358 (79%) sites for ex situ reefing by using simulations and rankings. If the offshore structures were to have remained in the sea at a headspace clearance of 30 m, 70 (15%) of the offshore structures were classified as high potential and 117 (26%) were classified as potential.  Figure 11 shows the calculated rank for each of the platforms based on governing factors considered in this study. It can be seen that most of the Rank 3 and 4 platforms were located in the Sabah and Sarawak region.

## 4. Conclusion

A spatial reef viability index was established based on seven parameters which are coral larval density, pelagic larval duration, sea currents, temperature, chlorophyll-a, depth, and substrate availability. A hydrodynamic model, coupled with particle tracking, was produced to match the pattern of larval scattering as well as drifting distance. All spatial and temporal analyses were mainly to identify optimal windows for coral settlement with monsoon effect being taken into consideration. Based on the integration of all parameters, the possibility of coral colonization in an area can be predicted. Therefore, a reefing method for the platform can be suggested to ensure reefing success in the South China Sea region. Based on the result, it was shown that 21% of the offshore platforms have potential to be artificial reef in the shallow tropical water of Malaysia. Meanwhile, remaining offshore platforms can be relocated to a high potential area indicated from the index map produced. This would eventually increase the reefing index of these lower rank platforms.

## Figures and Tables

**Table 1:** Coral seeding data from 8 sites.

Location	Latitude (N)	Longitude (E)	Total estimated coral larvae
Perhentian	5°55′55.8″	102°43′25.1″	25 161
Perhentian	5°55′31.0″	102°43′00.8″	200 032
Redang	5°46′22.77″	103°02′11.79″	2 126
Redang	5°44′44.87″	102°59′59.97″	26 330
Tioman	2°46′30.53″	104°13′12.92″	444 778
Tioman	2°48′34.34″	104°08′07.70″	605 142
TARP	06° 01.891′	116° 01.657′	10 035
TARP	06° 03.615′	116° 04.001′	12 267
Taman Laut Labuan, Pulau Kuraman	5°13′59.21″	115° 8′2.80″	5 910
Taman Laut Labuan, Pulau Kuraman	5°13′30.97″	115° 7′9.79″	21 376
Miri	4 20.583	113 53.900	494 816
Tanjung Kidurong	3.279974	113.007499	494 816
Similajau	3.55863	113.248818	494 816

## Data Availability

The data used to support the findings of this study are available from the corresponding author upon request.
